# Necessity and timing of angioplasty in acute large-vessel occlusion strokes due to intracranial atherosclerotic disease: A cohort analysis with data from the angel-ACT registry

**DOI:** 10.3389/fneur.2023.1087816

**Published:** 2023-03-16

**Authors:** Yiming Deng, Yunchu Yao, Xu Tong, Yue Yin, Anxin Wang, Yijun Zhang, Baixue Jia, Xiaochuan Huo, Gang Luo, Ning Ma, Dapeng Mo, Ligang Song, Xuan Sun, Feng Gao, Duanduan Chen

**Affiliations:** ^1^Department of Interventional Neuroradiology, Beijing Tiantan Hospital, Capital Medical University, Beijing, China; ^2^China National Clinical Research Center for Neurological Diseases, Beijing, China; ^3^Center of Stroke, Beijing Institute for Brain Disorders, Beijing, China; ^4^School of Life Science, Beijing Institute of Technology, Beijing, China; ^5^Department of Neurology, Beijing Tiantan Hospital, Capital Medical University, Beijing, China; ^6^School of Medical Technology, Beijing Institute of Technology, Beijing, China

**Keywords:** acute large-vessel occlusion strokes, intracranial atherosclerotic disease, efficacy, safety, angioplasty

## Abstract

**Background:**

The effects of angioplasty on intracranial atherosclerotic disease (ICAD)-related acute large-vessel occlusion stroke (LVOS) are unknown. We analyzed the efficacy and safety of angioplasty or stenting for ICAD-related LVOS and the optimal treatment duration.

**Methods:**

Patients with ICAD-related LVOS from a prospective cohort of the Endovascular Treatment Key Technique and Emergency Work Flow Improvement of Acute Ischemia Stroke registry were classified as follows: the early intraprocedural angioplasty and/or stenting (EAS) group was defined as the strategy using angioplasty or stenting without mechanical thrombectomy (MT) or one attempt of MT; the non-angioplasty and/or stenting (NAS) group, MT procedure without any angioplasty; and the late intraprocedural angioplasty and/or stenting (LAS) group, using same angioplasty techniques following two or more passes of MT. The primary endpoint was the modified Rankin Scale (mRS) score at 90 days. Other efficacy outcomes included mRS scores 0–1, mRS 0–2, and successful recanalization. Death within 90 days, and symptomatic ICH were safety endpoints. We use propensity score method to diminish the effect of treatment-selection bias. The odds ratio of recanalization rate and mRS score among EAS, NAS, and LAS groups were examined by unadjusted and adjusted logistic regression analysis among unweighted samples and inverse probability of treatment weighting (IPTW) samples.

**Results:**

We divided 475 cases into three groups. Functional outcomes at 90 days were better in the EAS group than in the NAS and LAS groups. The proportion of mRS 0–1, mRS 0–2, and successful recanalization cases were the highest in the EAS group. However, after IPTW, mortality rate among the three groups were similar (EAS vs. NAS vs. LAS: 19.0 vs. 18.1 vs. 18.7%, *p* = 0.98) as well as symptomatic intracranial hemorrhage within 24 h however, mortality rate and symptomatic intracranial hemorrhage among the three groups were similar. Logistic regression analysis in unweighted samples and IPTW samples both showed that EAS group had better outcomes. IPTW-adjusted logistic regression analysis demonstrated that the EAS group had better outcomes (mRS 0–1) than the NAS group (adjusted odds ratio [aOR], 0.55; 95% confidence interval [CI]: 0.34–0.88, *p* = 0.01) and LAS (aOR, 0.39; 95% CI: 0.22–0.68, *p* = 0.001).

**Conclusions:**

Angioplasty and/or stenting should be performed at an early stage for ICAD-related acute LVOS.

**Registration:**

URL: https://www.clinicaltrials.gov; Unique identifier: NCT03370939.

## Introduction

Mechanical thrombectomy (MT) is gaining popularity as the gold standard for the treatment of patients with acute large-vessel occlusion strokes (LVOS) owing to its efficacy in different stroke types (1–4). Compared with embolic etiologies, intracranial atherosclerotic disease (ICAD) remains a huge therapeutic challenge because MT alone does not effectively resolve the underlying atheromatous plaque, thus often requiring angioplasty or stenting as mechanical rescue treatment (5–7). Unfortunately, *in situ* atherothrombotic occlusions are more commonly encountered in non-white populations and patients with diabetes and hypertension (8, 9).

Previous studies have demonstrated the safety and efficacy of direct and emergency angioplasty, and/or stenting after thrombectomy in certain patients with ICAD-related LVOS (10–12). However, no conclusions have been attained regarding whether angioplasty should be used in ICAD-related LVOS. Here, we aimed to assess both the necessity and optimal timing of angioplasty or stenting for ICAD-related acute LVOS by describing the safety and efficacy of different endovascular strategies.

## Methods

### Study population

LVOS patients receiving MT were selected from the Endovascular Treatment Key Technique and Emergency Work Flow Improvement of Acute Ischemic Stroke (ANGEL-ACT) database (ClinicalTrials.gov Identifier: NCT 03370939), a prospective nationwide registry of 1,793 continuous patients in 111 hospitals from 26 provinces of China between November 2017 and March 2019 (13). Patients with ICAD-related LVOS were included in the registry. The exclusion criteria were as follows: (1) without EVT medical records; (2) without the TOAST classification appraisal; and (3) with small-vessel occlusion and/or cardioembolism without ICAD.

The study was approved by the Ethics Committees of the Beijing Tiantan Hospital and all participating centers. All procedures were conducted in accordance with the 1964 Declaration of Helsinki and subsequent amendments. Written informed consent from all patients or their legally authorized representatives was obtained.

### Data collection

Data on the baseline demographic characteristics (age and sex), medical history (hypertension, atrial fibrillation, diabetes mellitus, current smoking, antiplatelet agents, and anticoagulants), clinical characteristics (onset-to-door time, systolic blood pressure, Alberta Stroke Program Early Computed Tomography Score, National Institutes of Health Stroke Scale and intravenous thrombolysis), site of intracranial occlusion location, presence of tandem occlusion, type of anesthesia, and premorbid Modified Rankin Scale (mRS) scores were recorded.

### Endovascular treatment and classification of strategy

Either local anesthetic or general anesthetic was utilized for the procedure depending on the patient's cooperation and condition. If no contraindications were available, intravenous thrombolysis was performed before MT. After digital subtraction angiography, the neurointerventionist decided optimal strategy and materials for endovascular therapy.

The type of surgical strategy depends on the surgical situation and personal experience of neurointerventionists. Different strategies for *in situ* stenosis include balloon expansion angioplasty only (Gateway, Stryker, Kalamazoo, MI, USA; Neuro-RX SINOMED, Tianjin, China), balloon-mounted stents only (Apollo, MicroPort, Shanghai, China), balloon-mounted stents, or self-expanding stents (Wingspan or EZ, Stryker, Kalamazoo, MI, USA; Solitaire AB, Medtronic, Minneapolis, Minnesota, USA; Enterprise, Codman & Shurtleff Inc., Miami, FL, USA) after balloon expansion.

ICAD-related LVOS was divided into three groups based on the different treatment strategies. The early intraprocedural angioplasty and/or stenting (EAS) group was defined as the strategy involving balloon angioplasty alone, balloon-mounted stenting, or self-expanding stent after either no or one single pass of MT. The non-angioplasty and/or stenting (NAS) group was defined as undergoing the MT procedure without any angioplasty (including multiple passes of MT). Moreover, the late intraprocedural angioplasty and/or stenting (LAS) group was defined as the strategy of balloon angioplasty alone, balloon-mounted stenting, or self-expanding stent after two or more passes of MT.

### Outcome measures

Clinical outcomes included both efficacy and safety assessments, and all data were recorded by experienced investigators. We considered the functional outcome at 90-days post procedure (90-day mRS score) as the primary efficacy endpoint (Supplementary material). Meanwhile, mRS 0–1, mRS 0–2, and mRS 0–3, and successful recanalization—defined as the modified thrombolysis 2b/3 in cerebral infarction (14)—were considered as the secondary efficacy outcomes. Death within 90 days, symptomatic ICH were considered safety endpoints according to the Heidelberg Bleeding Classification (15). We also recorded procedure-related complications including intraprocedural embolization, arterial dissection, arterial perforation, and vasospasm requiring treatment.

### Statistical analysis

Data were recorded in standard forms and double-keyed into the EpiData statistics document. For continuous and ordinal variables, data are presented as medians (interquartile ranges [IQRs]), and for categorical variables, data are expressed as numbers (percentages). The student *t*-test was used for parametric data, while the non-parametric test (Mann–Whitney *U*-test) was used to compare the mean or median, respectively; Fisher's exact test or Pearson's chi-square test was used to compare the proportions or frequencies, respectively.

We used propensity scores to account for potential confounding factors and derive IPTW. The propensity score was estimated using a logistic regression model in which treatment assignment (EAS, NAS, and LAS) was regressed on the following covariates: demographic characteristics, hypertension, systolic blood pressure, atrial fibrillation, baseline NIHSS score, baseline ASPECTS score, presence of tandem occlusion, and type of anesthesia. Standardized mean differences were used to assess between-group balance of baseline characteristics, and a standardized mean difference smaller than 10% was considered insignificant difference.

We performed univariable logistic regression analysis, multivariable logistic regression analysis as well as IPTW-adjusted multivariable logistic regression analysis. The adjusted odds ratios (aOR) with corresponding 95% confidence intervals (CI) were determined using the multivariate logistic regression analysis to compare successful recanalization and clinical outcomes at 90 days between the three groups. We also evaluated the outcomes of EAS, NAS, and LAS groups with 1:1 propensity score matching using the nearest-neighbor method, however, the sample size was small and the results were inconclusive. The outcomes after propensity score matching differed significantly while *P*-value was still over 0.05 due to small sample size (Supplementary material). SAS Version 9.4 (SAS Institute, Cary, NC, USA) was used to perform statistical analysis.

## Results

### Baseline and procedural characteristics

**Figure 1:**
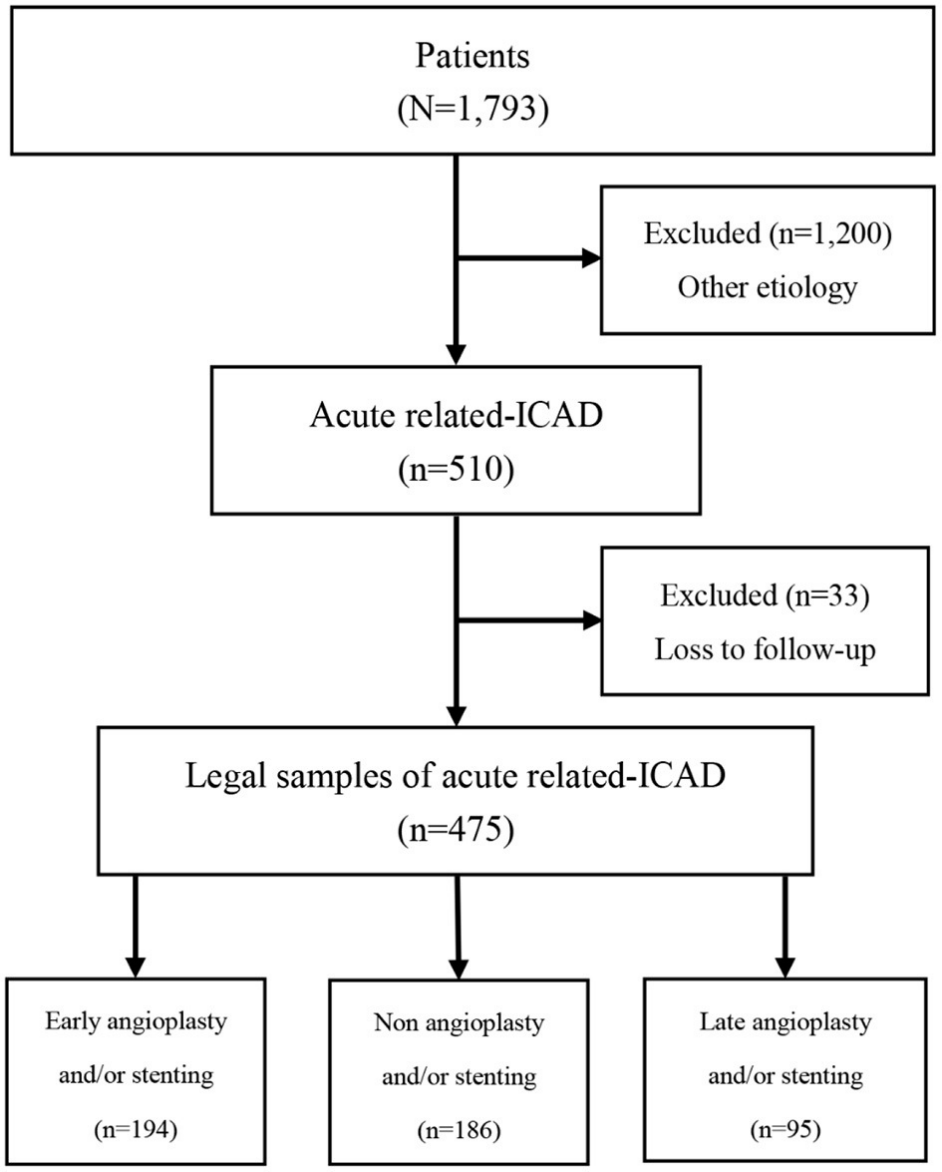
Flow diagram of recruitment of patients with acute intracranial atherosclerotic disease-related large-vessel occlusion stroke.

Table 1 showed unweighted and IPTW baseline characteristics of LVOS patients in EAS, NAS and LAS groups. Among the 1,793 participants in the Angel-ACT group, 475 cases met the inclusion criteria (27.9%) and were divided into three groups: 194 (40.1%) in the EAS group, 186 (40.3%) in the NAS group, and 95 (19.6%) in the LAS group. The process of patient selection is shown in Figure 1.

**Table 1:** Baseline characteristics of EAS, NAS, and LAS groups before and after inverse probability of treatment weighting^*^.

	**Unweighted sample (*****n*** = **475)**				**IPTW sample (*****n*** = **430.65)**			
**Variables**	**EAS (*****n*** = **194)**	**NAS (*****n*** = **186)**	**LAS (*****n*** = **95)**	**SMD**	**EAS (*****n*** = **150.15)**	**NAS (*****n*** = **187.55)**	**LAS (*****n*** = **92.95)**	**SMD**
**Demographic characteristics**								
Median age, y, median (IQR)	61 (54–67)	65 (54–72)	60 (51–68)	0.198	63 (54–68)	61 (52–69)	60 (53–67)	0.098
Men	154 (79.4)	137 (73.7)	83 (87.4)	0.234	32 (21.0)	38 (20.1)	18 (18.8)	0.036
**Medical history**								
Hypertension	144 (74.2)	113 (60.8)	55 (57.9)	0.211	91 (60.5)	120 (63.8)	59 (62.9)	0.045
Diabetes	41 (21.1)	41 (22.0)	22 (23.2)	0.067	25 (16.4)	42 (22.6)	22 (23.8)	0.123
Atrial fibrillation	6 (3.1)	31 (16.7)	6 (6.3)	0.306	18 (12.0)	18 (9.6)	9 (9.4)	0.054
Smoking	99 (51.0)	91 (48.9)	46 (48.4)	0.080	73 (48.4)	97 (51.8)	38 (41.1)	0.145
Antiplatelet agents	40 (20.6)	30 (16.1)	14 (14.7)	0.029	24 (16.2)	32 (17.0)	12 (13.1)	0.073
Anticoagulants	5 (2.6)	3 (1.6)	1 (1.1)	0.108	6 (4.1)	4 (2.1)	1 (0.8)	0.145
**Clinical characteristics**								
Onset-to-door time, min, median (IQR)	178 (77–340)	180 (80–330)	180 (88–341)	0.005	170 (61–294)	180 (70–330)	148 (57–284)	0.073
SBP, mmHg, median (IQR)	150 (137–168)	145 (130–160)	150 (132–172)	0.208	146 (132–164)	149 (132–165)	149 (130–164)	0.032
Baseline NIHSS score, median (IQR)	14 (8–20)	16 (12–22)	16 (11–21)	0.084	17 (10–22)	16 (12–22)	15 (11–21)	0.090
ASPECTS, median (IQR)	8 (7–10)	9 (7–10)	8 (7–10)	0.109	8 (7–10)	8 (7–10)	8 (7–10)	0.033
IV thrombolysis before procedure	53 (27.3)	36 (19.4)	22 (23.2)	0.175	46 (30.6)	36 (19.1)	23 (24.4)	0.179
Intracranial occlusion location				0.280				0.296
ICA	42 (21.7)	39 (21.0)	16 (16.8)		25 (16.4)	39 (20.9)	13 (14.0)	
M1	81 (41.8)	84 (45.2)	39 (41.1)		68 (44.9)	85 (45.1)	44 (47.7)	
M2	7 (3.6)	13 (7.0)	2 (2.1)		9 (5.9)	12 (6.2)	1 (1.1)	
VA	63 (32.5)	45 (24.2)	36 (37.9)		48 (32.0)	47 (25.1)	33 (35.2)	
Other	1 (0.5)	5 (2.7)	2 (2.1)		1 (0.8)	5 (2.7)	2 (2.0)	
Presence of tandem occlusion	49 (25.3)	27 (14.5)	22 (23.2)	0.148	27 (18.3)	37 (19.8)	17 (18.5)	0.026
Type of anesthesia				0.314				0.086
Local anesthesia only	81 (41.8)	86 (46.2)	24 (25.3)		58 (38.3)	74 (39.3)	32 (33.9)	
Local anesthesia plus sedation	84 (43.3)	75 (40.3)	50 (52.6)		67 (44.5)	81 (43.3)	46 (49.5)	
General anesthesia	29 (15.0)	25 (13.4)	21 (22.1)		26 (17.2)	33 (17.5)	15 (16.6)	
Premorbid mRS score				0.149				0.151
0	160 (82.5)	163 (87.6)	79 (84.0)		128 (85.4)	163 (87.1)	78 (83.9)	
1	30 (15.5)	23 (12.4)	13 (13.8)		19 (12.7)	24 (12.9)	14 (14.9)	
2	4 (2.1)	0 (0.0)	2 (2.1)		3 (2.0)	0 (0.0)	1 (1.2)	

^*^The numbers of patients in IPTW samples are not necessarily integers due to inverse probability weighting.

EAS, early angioplasty and/or stenting (n = 201); LAS, late angioplasty and/or stenting (n = 98); NAS, non-angioplasty and/or stenting (n = 202); IPTW, inverse probability of treatment weighting; IQR, interquartile range; SMD, standardized mean difference; SBP, systolic blood pressure; NIHSS, National Institutes of Health Stroke Scale; ASPECTS, Alberta Stroke program Early CT score; ICA, internal carotid artery; M1, M1 segment of the middle cerebral artery; M2, M2 segment of the middle cerebral artery; VA, vertebral artery; mRS, modified Rankin Scale.

The three groups showed baseline differences in age, sex, preoperative systolic blood pressure, preoperative National Institutes of Health Stroke Scale (NIHSS) score, Alberta Stroke Program Early CT Score (ASPECTS), presence of tandem stenosis, and type of anesthesia (Table 1). Generally, patients in the EAS (mean age: 61 years) and LAS (mean age: 60 years) groups were younger than those in the NAS group (mean age: 65 years), overall *p* = 0.010; EAS group vs. NAS group: *p* = 0.01; EAS group vs. LAS group: *p* = 0.65; and NAS group vs. LAS group: *p* = 0.02 (*p*-value was shown in Supplementary material). The EAS (3.1%) and LAS (6.3%) groups had a lower proportion of atrial fibrillation than the NAS group (16.7%), overall *p* < 0.0001; EAS group vs. NAS group, *p* < 0.0001; EAS group *vs*. LAS group, *p* = 0.20; NAS group vs. LAS group, *p* = 0.02. The EAS group had lower NIHSS (EAS group: 14 vs. NAS group: 16 vs. LAS group: 16; overall *p* = 0.02; EAS group vs. NAS group: *p* = 0.01; EAS group vs. LAS group: *p* = 0.05; NAS group vs. LAS group: *p* = 0.81) and ASPETS (EAS group: 8 vs. NAS group: 9 vs. LAS group: 8; overall *p* = 0.03; EAS group vs. NAS group: *p* = 0.01; EAS group vs. LAS group: *p* = 0.81; NAS group vs. LAS group: *p* = 0.08) than NAS group and higher systolic blood pressure (EAS group: 150 mmHg vs. NAS group: 145 mmHg vs. NAS group: 150 mmHg; overall *p* = 0.02; EAS group vs. NAS group: *p* = 0.01; EAS group vs. LAS group: *p* = 0.74; NAS group vs. LAS group: *p* = 0.07) than NAS group. The EAS group also had a higher proportion of tandem occlusions than the NAS group (EAS group: 25.3% vs. NAS group: 14.5% vs. NAS group: 23.2%; overall *p* = 0.03; EAS group vs. NAS group: *p* = 0.01; EAS group vs. LAS group: *p* = 0.70; NAS group vs. LAS group: *p* = 0.07).

The standardized mean differences (SMD) of baseline characteristics of patients showed that the EAS, NAS, and LAS groups differed in terms of demographic characteristics, medical history, clinical characteristics, intracranial occlusion location, type of anesthesia, and premorbid mRS score before inverse probability of treatment weighting. We conducted IPTW to account for confounding factors, and the baseline variables are more balanced after IPTW. However, the SMD of diabetes, smoking, anticoagulants, IV thrombolysis before procedure, intracranial occlusion location and premorbid mRS score are still over 0.1.

### Outcomes measures

Clinical outcome assessments, including efficacy and safety assessments, significantly differed among the three groups (Table 2).

**Table 2:** Comparison of treatment effect of EAS, NAS, and LAS groups.

**Parameter**	**mTICI 2b-3**		**(mRS 0–1) at 90 d**		**(mRS 0–2) at 90 d**		**Mortality at 90 d**	
	**OR (95% CI)**	***P***	**OR (95% CI)**	***P***	**OR (95% CI)**	***P***	**OR (95% CI)**	***P***
**Unadjusted estimates**								
NAS vs. EAS	0.39 (0.15–1.04)	0.06	0.56 (0.38–0.85)	0.006	0.54 (0.36–0.81)	0.003	1.84 (1.03–3.31)	0.04
LAS vs. EAS	0.31 (0.11–0.88)	0.03	0.40 (0.24–0.67)	0.001	0.44 (0.26–0.72)	0.001	2.06 (1.05–4.05)	0.04
**Adjusted estimates** ^*^								
NAS vs. EAS	0.40 (0.14–1.12)	0.08	0.54 (0.34–0.86)	0.009	0.54 (0.34–0.85)	0.01	1.60 (0.84–3.05)	0.15
LAS vs. EAS	0.28 (0.09–0.83)	0.02	0.41 (0.23–0.72)	0.002	0.46 (0.27–0.80)	0.01	1.90 (0.93–3.89)	0.08
**IPTW adjusted estimates** ^*^^†^								
NAS vs. EAS	0.25 (0.06–0.78)	0.03	0.55 (0.34–0.88)	0.01	0.54 (0.34–0.85)	0.01	1.08 (0.60–1.99)	0.79
LAS vs. EAS	0.16 (0.04–0.53)	0.01	0.39 (0.22–0.68)	0.001	0.45 (0.25–0.78)	0.004	1.25 (0.61–2.55)	0.54

mTICI, modified thrombolysis in cerebral infarction; OR, odds ratio; NIHSS, National Institutes of Health Stroke Scale; mRS, modified Rankin Scale. Early angioplasty and/or stenting group was used as the reference category.

^*^Adjusted for age, sex, hypertension, systolic blood pressure, atrial fibrillation, baseline NIHSS score, baseline ASPECTS score as a continuous variable, presence of tandem occlusion, and type of anesthesia.

^†^IPTW obtained after adjustment.

The safety assessments were similar among EAS, NAS, and LAS groups. Mortality rate among the three groups were similar (EAS vs. NAS vs. LAS: 19.0 vs. 18.1 vs. 18.7%, *p* = 0.98) as well as symptomatic intracranial hemorrhage within 24 h (EAS vs. NAS vs. LAS: 9.7 vs. 2.3 vs. 9.7%, *p* = 0.11; Supplementary material).

After adjusting for age, sex, hypertension, atrial fibrillation, systolic blood pressure, baseline NIHSS score, baseline ASPECTS score as a continuous variable, presence of tandem occlusion, and type of anesthesia, logistic regression analyses revealed that the EAS group had better outcomes at 90 days than those of the NAS and LAS groups (mRS 0–1: EAS group vs. NAS group, aOR, 0.54, 95% CI: 0.34–0.86, *p* = 0.009; EAS group vs. LAS group, aOR, 0.41, 95% CI: 0.23–0.72, *p* = 0.002; mRS 0–2: EAS group vs. NAS group, aOR, 0.54, 95% CI: 0.34–0.85, *p* = 0.01; EAS group vs. LAS group, aOR, 0.46, 95% CI: 0.27–0.80, *p* = 0.01). The recanalization rate was higher in the EAS than in the LAS group (EAS group vs. LAS group, aOR, 0.28; 95% CI: 0.09–0.83, *p* = 0.02; Table 2). And IPTW-adjusted logistic regression model showed more distinguished outcome of EAS group which justified our conclusion (mRS 0–1: EAS group vs. NAS group, aOR, 0.55, 95% CI: 0.34–0.88, *p* = 0.01; EAS group vs. LAS group, aOR, 0.39, 95% CI: 0.22–0.68, *p* = 0.001; mRS 0–2: EAS group vs. NAS group, aOR, 0.54, 95% CI: 0.34–0.85, *p* = 0.01; EAS group vs. LAS group, aOR, 0.45, 95% CI: 0.25–0.78, *p* = 0.004). The recanalization rate was higher in the EAS than in the LAS group (EAS group vs. LAS group, aOR, 0.16; 95% CI: 0.04–0.53, *p* = 0.02; Table 2).

## Discussion

To our knowledge, this is the first study to explore the association between different endovascular treatment strategies for angioplasty and functional prognosis in ICAD-related LVOS. This study revealed two main findings as follows: (1) performing angioplasty and/or stenting in patients with acute ICAD-related LVOS compared to patients without angioplasty is effective and safe; and (2) EAS is superior to LAS.

First, our results suggest that angioplasty yields greater benefits than does the choice to not undergo angioplasty. EAS had better revascularization rates than NAS groups on the final angiogram according to IPTW-adjusted logistic regression analysis. Even with a longer time for door to revascularization, the EAS group still exhibited better outcomes. However, the complication rate is low. Previous retrospective studies have identified better results with the performance of angioplasty (11, 16, 17). One retrospective study confirmed that angioplasty and/or stenting could be as the first-line treatment strategy for patients with acute anterior large-vessel occlusion caused by atherosclerosis (18). We considered urgent angioplasty and/or stenting to be feasible for the following reasons. Blood flow conditions can be maintained after angioplasty treatment (16). The possible causes of acute LVOS due to *in situ* stenosis include *in situ* thrombosis and proximal cardiogenic or arterial-to-arterial embolus incarceration in the stenosis (19). Thrombosis *in situ*, if the injured endothelium is not treated with angioplasty, may repeatedly lead to neovascularization and proximal embolus, if not relieved of the cause, will still be dislodged and lead to re-occlusion. Therefore, angioplasty can reduce re-occlusion in these patients. Second, angioplasty does not increase the risk of hyperperfusion bleeding (18). Finally, re-occlusion may occur immediately even in cases where thrombolysis is successful, as fibrinolytic agents may exacerbate the prothrombotic tendency of atherosclerotic lesions.

This study also elaborates on the optimal timing for first time angioplasty. Our results reveal that the outcomes for angioplasty treatment, either without MT or after one attempt pass of MT, is better than that after two or more MTs, with mortality reduced by half. This may be because multiple MTs may lead to more severe vascular endothelial injury, increasingly poorer outcomes, and significantly lower recanalization rates. After multiple thrombectomies, the operative time was significantly delayed, and prognosis worsened. Simultaneously, more attempts at MT may lead to vascular injury and significantly increased bleeding rates—resulting in poor prognosis and increased mortality. Therefore, early angioplasty can achieve more significant clinical outcomes than late angioplasty. A study showed that in ICAD populations, angioplasty and stenting had better efficacy than stent-retriever (18). Unfortunately, no studies reported satisfactory results when performing thrombectomy. After a single thrombectomy, the embolus was clearly identified as an *in-situ* stenosis. However, multiple thrombectomy passes causes negative effects such as vascular plaque exposure, intimal damage, and vasospasm.

Although MT has become the standard treatment for acute intracranial arterial occlusion (20, 21), the treatment for patients with ICAD is different from that for embolization. Therefore, it is important to identify ICAD early. Although ICAD is consistently associated with advanced age, the risk of ICAD in young people should not be ignored (22). Vascular risk factors for ICAD—including hypertension (23), hypercholesterolemia, diabetes, and smoking—Gutierrez et al. (24) can increase the patient's risk. Thus, timely detection and treatment of vascular risk factors is necessary to prevent further disease development. ICAD diagnostic methods include routine cerebral angiography, CT angiography (CTA), magnetic resonance angiography, high-resolution MRI, and transcranial Doppler ultrasound. Notably, MRI-based high-resolution imaging can directly show state of the intracranial arterial wall. Using these diagnostic imaging techniques can help identify high-risk populations. However, CTA has higher specificity and sensitivity for detecting ICAD and is now the method of choice for diagnosing ICAD in the United States (8). Briefly, ICAD should be identified, and angioplasty should be administered as early as possible.

In summary, angioplasty should be performed as early as possible in ICAD-related LOVS. It is essential to identify ICAD lesions before or after one MT pass. Current methods include the first-pass effect (25) and artificial intelligence (AI) technology (26). Once an ICAD lesion has been identified, thrombectomy should be performed according to the specific situation. MT should be performed first to remove the thrombus surrounding the *in-situ* stenosis. We also confirmed the presence and morphology of the stenosis based on the shape of the stent. This study used the STROBE cohortreporting guidelines.

This study had some limitations. Firstly, it was not a randomized study and thus can only partly illustrate the issue. Secondly, owing to the small, homogenous sample size and the prevalence of ICAD in China, our results may not be generalizable. Thus, future studies conducted on a wider scale with more data are required to confirm our results. Finally, although our results are controversial, angioplasty undeniably holds promise in select patients.

In conclusion, angioplasty and/or stenting is effective and safe and should be performed at an early stage of ICAD-related acute LVOS. However, randomized controlled trials are required to confirm this hypothesis.

## Data availability statement

The original contributions presented in the study are included in the article/Supplementary material, further inquiries can be directed to the corresponding author/s.

## Ethics statement

The studies involving human participants were reviewed and approved by IRB of Beijing Tiantan Hospital, Capital Medical University. The patients/participants provided their written informed consent to participate in this study.

## Author contributions

YD and YYa participated in the research design, model computations, data analysis, and drafted the manuscript. XT, YYi, AW, and YZ carried out the data collection and computation. GL, NM, and FG participated in model computations. DM, LS, and XS participated in table and figure design. BJ and XH participated in data analysis. DC and FG supervised this work, carried out the research design, and revised the manuscript. All authors contributed to the article and approved the submitted version.
