# Supplementary material for: Necessity and timing of angioplasty in acute large-vessel occlusion strokes due to intracranial atherosclerotic disease: A cohort analysis with data from the angel-ACT registry
Source: Front Neurol. 2023 Mar 16;14:1087816. doi: 10.3389/fneur.2023.1087816 (PMC10062386; doi:10.3389/fneur.2023.1087816)
Supplement: Supplementary file 1 [file Data_Sheet_1.docx]

**Supplementary Table 1.** Comparison of the baseline characteristics between EAS, NAS, and LAS groups after propensity score matching(N=138).

| Variables | EAS（n=46） | NAS（n=46） | LAS（n=46） | P value | | | |
| --- | --- | --- | --- | --- | --- | --- | --- |
|  |  |  |  | Overall | EAS  vs  NAS | EAS  vs  LAS | NAS  vs  LAS |
| Demographic characteristics | | | | | | | |
| Median age, y, median (IQR) | 59.00 (53.00–69.00) | 62.50 (52.00–69.00) | 63.50 (55.00–69.00) | 0.63 | 0.44 | 0.38 | 0.99 |
| Men | 38 (82.61) | 38 (82.61) | 38 (82.61) | 1.00 | 1.00 | 1.00 | 1.00 |
| Medical history | | | | | | | |
| Hypertension | 28 (60.87) | 32 (69.57) | 32 (69.57) | 0.59 | 0.38 | 0.38 | 1.00 |
| Diabetes | 7 (15.22) | 15 (32.61) | 11 (23.91) | 0.15 | 0.05 | 0.29 | 0.35 |
| Atrial fibrillation | 1 (2.17) | 1 (2.17) | 3 (6.52) | 0.44 | 1.00 | 0.31 | 0.31 |
| Smoking | 28 (60.87) | 20 (43.48) | 16 (34.78) | 0.04 | 0.10 | 0.01 | 0.39 |
| Antiplatelet agents | 8 (17.39) | 10 (21.74) | 6 (13.04) | 0.55 | 0.60 | 0.56 | 0.27 |
| Anticoagulants | 1 (2.17) | 1 (2.17) | 1 (2.17) | 1.00 | 1.00 | 1.00 | 1.00 |
| Clinical characteristics | | | | | | | |
| Onset-to-door time, min, median (IQR) | 189.00 (75.00–339.00) | 180.00 (60.00–330.00) | 137.00 (40.00–300.00) | 0.63 | 0.86 | 0.37 | 0.46 |
| SBP, mmHg, median (IQR) | 143.00 (135.00–170.00) | 150.00 (136.00–167.00) | 150.00 (133.00–164.00) | 0.97 | 0.87 | 0.93 | 0.81 |
| Baseline NIHSS score, median (IQR) | 16.50 (10.00–20.00) | 16.00 (10.00–22.00) | 17.00 (11.00–23.00) | 0.65 | 0.84 | 0.36 | 0.52 |
| ASPECTS, median (IQR) | 8.00 (7.00–10.00) | 8.00 (6.00–10.00) | 8.00 (7.00–10.00) | 0.59 | 0.31 | 0.59 | 0.62 |
| IV thrombolysis before procedure | 11 (23.91) | 3 (6.52) | 9 (19.57) | 0.07 | 0.02 | 0.61 | 0.06 |
| Intracranial occlusion location | | | | 0.18 | 0.28 | 0.36 | 0.15 |
| ICA | 10 (21.74) | 5 (10.87) | 8 (17.39) |  |  |  |  |
| M1 | 23 (50.00) | 22 (47.83) | 19 (41.30) |  |  |  |  |
| M2 | 1 (2.17) | 4 (8.70) | 0 (0.00) |  |  |  |  |
| VA | 12 (26.09) | 15 (32.61) | 19 (41.30) |  |  |  |  |
| Other | 0 (0.00) | 0 (0.00) | 0 (0.00) |  |  |  |  |
| presence of tandem occlusion | 11 (23.91) | 6 (13.04) | 6 (13.04) | 0.27 | 0.18 | 0.18 | 1.00 |
| Type of anesthesia | | | | 0.77 | 0.91 | 0.44 | 0.65 |
| Local anesthesia only | 18 (39.13) | 17 (36.96) | 13 (28.26) |  |  |  |  |
| Local anesthesia plus sedation | 20 (43.48) | 22 (47.83) | 26 (56.52) |  |  |  |  |
| General anesthesia | 8 (17.39) | 7 (15.22) | 7 (15.22) |  |  |  |  |
| Premorbid mRS score | | | | 0.18 | 0.13 | 0.24 | 0.56 |
| 0 | 33 (71.74) | 40 (86.96) | 38 (82.61) |  |  |  |  |
| 1 | 11 (23.91) | 6 (13.04) | 8 (17.39) |  |  |  |  |
| 2 | 2 (4.35) | 0 (0.00) | 0 (0.00) |  |  |  |  |

Abbreviations: EAS, early angioplasty and/or stenting (n=201); LAS, late angioplasty and/or stenting (n=98); NAS, non-angioplasty and/or stenting (n=202); IPTW, inverse probability of treatment weighting; IQR, interquartile range; SMD, standardized mean difference; SBP, systolic blood pressure; NIHSS, National Institutes of Health Stroke Scale; ASPECTS, Alberta Stroke program Early CT score; ICA, internal carotid artery; M1, M1 segment of the middle cerebral artery; M2, M2 segment of the middle cerebral artery; VA, vertebral artery; mRS, modified Rankin Scale.

**Supplementary Table 2.** Summary of results of Primary outcomes, Secondary outcomes, Safety outcomes, Procedure-related complications after propensity score matching(N=138).

| Outcomes | EAS（n=46） | NAS（n=46） | LAS（n=46） | *P* value | | | |
| --- | --- | --- | --- | --- | --- | --- | --- |
|  |  |  |  | Overall | EAS  *vs*  NAS | EAS  *vs*  LAS | NAS  *vs*  LAS |
| Primary outcome | | | | | | | |
| mRS at 90 d, median (IQR) | 1.00 (0.00–4.00) | 3.00 (0.00–5.00) | 4.00 (0.00–5.00) | 0.03 | 0.15 | 0.01 | 0.26 |
| Secondary outcomes | | | | | | | |
| mRS 0–1 at 90 d | 26 (56.52) | 20 (43.48) | 14 (30.43) | 0.04 | 0.21 | 0.01 | 0.20 |
| mRS 0–2 at 90 d | 28 (60.87) | 21 (45.65) | 17 (36.96) | 0.07 | 0.14 | 0.02 | 0.40 |
| mRS 0–3 at 90 d | 31 (67.39) | 25 (54.35) | 21 (45.65) | 0.11 | 0.20 | 0.04 | 0.40 |
| Change in NIHSS score at 24 h, median (IQR)§ | -3.00 (-6.00–0.00) | -1.00 (-5.00–0.00) | -2.00 (-5.00–0.00) | 0.30 | 0.14 | 0.26 | 0.70 |
| Change in NIHSS score at 7 d, median (IQR)¶ | -9.00 (-13.00–-3.00) | -3.50 (-7.50–0.00) | -5.00 (-11.00–0.00) | 0.03 | 0.01 | 0.05 | 0.68 |
| Successful recanalization at final angiogram | 44 (95.65) | 43 (93.48) | 41 (89.13) | 0.47 | 0.65 | 0.24 | 0.46 |
| Door-to-puncture time, min, median (IQR) | 122.50 (85.00–200.00) | 124.00 (84.00–183.00) | 140.00 (100.00–170.00) | 0.79 | 0.65 | 0.71 | 0.53 |
| Puncture-to-recanalization time, min, median (IQR) | 93.50 (53.00–154.00) | 87.00 (50.00–132.00) | 118.00 (89.00–184.00) | 0.01 | 0.30 | 0.05 | 0.005 |
| Pass number of thrombectomy, median (IQR) | 1.00 (1.00–1.00) | 1.00 (1.00–2.00) | 2.00 (2.00–3.00) | <0.0001 | <0.0001 | <0.0001 | <0.0001 |
| Safety outcomes | | | | | | | |
| Death within 90 d | 3 (6.52) | 5 (10.87) | 8 (17.39) | 0.26 | 0.46 | 0.11 | 0.37 |
| Any ICH within 24 h | 7 (15.22) | 8 (17.39) | 11 (24.44) | 0.50 | 0.78 | 0.27 | 0.41 |
| Symptomatic ICH within 24 h🞲🞲(Heidb) | 1 (2.17) | 0 (0.00) | 3 (6.67) | 0.18 | 0.31 | 0.30 | 0.07 |
| Procedure-related complications | | | | | | | |
| Intraprocedural embolization | 1 (2.17) | 1 (2.17) | 4 (8.70) | 0.21 | 1.00 | 0.17 | 0.17 |
| Arterial perforation | 2 (4.35) | 1 (2.17) | 2 (4.35) | 0.81 | 0.56 | 1.00 | 0.56 |
| Arterial dissection | 2 (4.35) | 0 (0.00) | 2 (4.3) | 0.36 | 0.15 | 1.00 | 0.15 |
| Vasospasm Requiring treatment | 0 (0.00) | 1 (2.17) | 0 (0.00) | 0.37 | 0.31 | - | 0.31 |

Abbreviations: EAS, early angioplasty and/or stenting; NAS, non-angioplasty and/or stenting; LAS, late angioplasty and/or stenting; NIHSS, National Institutes of Health Stroke Scale; mRS, modified Rankin Scale; ICH, intracranial hemorrhage; IQR, interquartile range.

**Supplementary Table 3.** Comparison of treatment effect of EAS, NAS, and LAS groups after propensity score matching(N=138).

| Parameter | mTICI 2b-3 | | (mRS 0-1) at 90 d | | (mRS 0–2) at 90 d | | Mortality at 90 d | |
| --- | --- | --- | --- | --- | --- | --- | --- | --- |
|  | OR (95% CI) | *P* | OR (95% CI) | *P* | OR (95% CI) | *P* | OR (95% CI) | *P* |
| Model 1* | | | | | | | | |
| NAS *vs.* EAS | 0.67 (0.11–3.99) | 0.66 | 0.59 (0.26–1.36) | 0.21 | 0.51 (0.21–1.22) | 0.13 | 1.75 (0.39–7.84) | 0.46 |
| LAS *vs.* EAS | 0.40 (0.08–2.06) | 0.66 | 0.34 (0.14–0.81) | 0.02 | 0.35 (0.14–0.86) | 0.02 | 3.09 (0.75–12.77) | 0.12 |

mTICI, modified thrombolysis in cerebral infarction; OR, odds ratio; NIHSS, National Institutes of Health Stroke Scale; mRS, modified Rankin Scale. Early angioplasty and/or stenting group was used as the reference category.

***Model 1:** unadjusted.

**Supplementary Table 4.** Comparison of the baseline characteristics between EAS, NAS, and LAS groups (N=475)

| Variables | EAS（n=194） | NAS（n=186） | LAS（n=95） | P value | | | |
| --- | --- | --- | --- | --- | --- | --- | --- |
|  |  |  |  | Overall | EAS  vs  NAS | EAS  vs  LAS | NAS  vs  LAS |
| Demographic characteristics | | | | | | | |
| Median age, y, median (IQR) | 61 (54-67) | 65 (54-72) | 60 (51-68) | 0.01 | 0.01 | 0.65 | 0.02 |
| Men | 154 (79.38) | 137 (73.66) | 83 (87.37) | 0.03 | 0.19 | 0.10 | 0.01 |
| Medical history | | | | | | | |
| Hypertension | 144 (74.23) | 113 (60.75) | 55 (57.89) | 0.004 | 0.01 | 0.005 | 0.64 |
| Diabetes | 41 (21.13) | 41 (22.04) | 22 (23.16) | 0.92 | 0.83 | 0.70 | 0.83 |
| Atrial fibrillation | 6 (3.09) | 31 (16.67) | 6 (6.32) | <0.0001 | <0.0001 | 0.20 | 0.02 |
| Smoking | 99 (51.03) | 91 (48.92) | 46 (48.42) | 0.89 | 0.68 | 0.68 | 0.94 |
| Antiplatelet agents | 40 (20.62) | 30 (16.13) | 14 (14.74) | 0.36 | 0.26 | 0.23 | 0.76 |
| Anticoagulants | 5 (2.58) | 3 (1.61) | 1 (1.05) | 0.63 | 0.51 | 0.39 | 0.71 |
| Clinical characteristics | | | | | | | |
| Onset-to-door time, min, median (IQR) | 177.5 (76.5-340) | 180 (80-330) | 180 (88-341) | 0.95 | 0.76 | 0.97 | 0.85 |
| SBP, mmHg, median (IQR) | 150 (137-168) | 145 (130-160) | 150 (132-172) | 0.02 | 0.01 | 0.74 | 0.07 |
| Baseline NIHSS score, median (IQR) | 14 (8-20) | 16 (12-22) | 16 (11-21) | 0.02 | 0.01 | 0.05 | 0.81 |
| ASPECTS, median (IQR) | 8 (7-10) | 9 (7-10) | 8 (7-10) | 0.03 | 0.01 | 0.81 | 0.08 |
| IV thrombolysis before procedure | 53 (27.32) | 36 (19.35) | 22 (23.16) | 0.19 | 0.07 | 0.45 | 0.46 |
| Intracranial occlusion location | | | | 0.14 | 0.11 | 0.51 | 0.10 |
| ICA | 42 (21.65) | 39 (20.97) | 16 (16.84) |  |  |  |  |
| M1 | 81 (41.75) | 84 (45.16) | 39 (41.05) |  |  |  |  |
| M2 | 7 (3.61) | 13 (6.99) | 2 (2.11) |  |  |  |  |
| VA | 63 (32.47) | 45 (24.19) | 36 (37.89) |  |  |  |  |
| Other | 1 (0.52) | 5 (2.69) | 2 (2.11) |  |  |  |  |
| presence of tandem occlusion | 49 (25.26) | 27 (14.52) | 22 (23.16) | 0.03 | 0.01 | 0.70 | 0.07 |
| Type of anesthesia | | | | 0.01 | 0.67 | 0.02 | 0.002 |
| Local anesthesia only | 81 (41.75) | 86 (46.24) | 24 (25.26) |  |  |  |  |
| Local anesthesia plus sedation | 84 (43.30) | 75 (40.32) | 50 (52.63) |  |  |  |  |
| General anesthesia | 29 (14.95) | 25 (13.44) | 21 (22.11) |  |  |  |  |
| Premorbid mRS score | | | | 0.30 | 0.09 | 0.94 | 0.13 |
| 0 | 160 (82.47) | 163 (87.63) | 79 (84.04) |  |  |  |  |
| 1 | 30 (15.46) | 23 (12.37) | 13 (13.83) |  |  |  |  |
| 2 | 4 (2.06) | 0 (0.00) | 2 (2.13) |  |  |  |  |

Abbreviations: EAS, early angioplasty and/or stenting (n=201); LAS, late angioplasty and/or stenting (n=98); NAS, non-angioplasty and/or stenting (n=202); IPTW, inverse probability of treatment weighting; IQR, interquartile range; SMD, standardized mean difference; SBP, systolic blood pressure; NIHSS, National Institutes of Health Stroke Scale; ASPECTS, Alberta Stroke program Early CT score; ICA, internal carotid artery; M1, M1 segment of the middle cerebral artery; M2, M2 segment of the middle cerebral artery; VA, vertebral artery; mRS, modified Rankin Scale.

**Supplementary Table 5.** Summary of results of Primary outcomes, Secondary outcomes, Safety outcomes, Procedure-related complications after inverse probability of treatment weighting (N=430.65).

| Variables | EAS（n=150.15） | NAS（n=187.55） | LAS（n=92.95） | P value |  |
| --- | --- | --- | --- | --- | --- |
|  |  |  |  | Overall | SMD |
| mRS at 90 d, median (IQR) | 1.00 (0.00, 5.00) | 3.00 (0.00, 5.00) | 3.00 (0.00, 5.00) | 0.30 | 0.171 |
| mRS 0–1 at 90 d | 79.6 (53.0) | 78.8 (42.0) | 32.2 (34.7) | 0.04 | 0.249 |
| mRS 0–2 at 90 d | 83.0 (55.3) | 81.4 (43.4) | 36.6 (39.3) | 0.06 | 0.215 |
| mRS 0–3 at 90 d | 93.5 (62.3) | 101.8 (54.3) | 48.5 (52.2) | 0.32 | 0.137 |
| Change in NIHSS score at 24 h, median (IQR)§ | -2.00 (-6.00, 0.00) | -1.00 (-5.00, 0.00) | -1.00 (-4.00, 0.00) | 0.52 | 0.055 |
| Change in NIHSS score at 7 d, median (IQR)¶ | -8.00 (-14.00, -2.00) | -6.00 (-11.08, -1.00) | -5.00 (-11.00, -2.00) | 0.41 | 0.107 |
| Successful recanalization at final angiogram | 146.9 (97.8) | 172.7 (92.1) | 82.0 (88.2) | 0.03 | 0.259 |
| Door-to-puncture time, min, median (IQR) | 140.60 (85.00, 215.12) | 116.50 (75.65, 180.29) | 128.00 (86.57, 180.32) | 0.57 | 0.033 |
| Puncture-to-recanalization time, min, median (IQR) | 102.22 (55.00, 149.94) | 88.16 (54.49, 130.99) | 121.86 (89.81, 186.53) | <0.001 | 0.406 |
| Pass number of thrombectomy, median (IQR) | 1.00 (1.00, 1.00) | 2.00 (1.00, 2.00) | 2.00 (2.00, 4.00) | <0.001 | 1.096 |
| Death within 90 d | 28.5 (19.0) | 33.9 (18.1) | 17.4 (18.7) | 0.98 | 0.015 |
| Any ICH within 24 h | 25.6 (17.9) | 29.4 (15.9) | 24.9 (27.8) | 0.20 | 0.194 |
| Symptomatic ICH within 24 h🞲🞲(Heidb) | 13.9 ( 9.7) | 4.2 ( 2.3) | 8.7 ( 9.7) | 0.11 | 0.211 |
| Intraprocedural embolization | 5.1 ( 3.4) | 7.2 ( 3.8) | 5.2 ( 5.6) | 0.72 | 0.071 |
| Arterial perforation | 3.0 ( 2.0) | 2.2 ( 1.2) | 2.0 ( 2.2) | 0.81 | 0.054 |
| Arterial dissection | 3.5 ( 2.3) | 1.9 ( 1.0) | 2.7 ( 2.9) | 0.55 | 0.094 |
| Vasospasm Requiring treatment | 2.0 ( 1.3) | 2.3 ( 1.2) | 0.7 ( 0.7) | 0.89 | 0.040 |

Abbreviations: EAS, early angioplasty and/or stenting; NAS, non-angioplasty and/or stenting; LAS, late angioplasty and/or stenting; NIHSS, National Institutes of Health Stroke Scale; mRS, modified Rankin Scale; ICH, intracranial hemorrhage; IQR, interquartile range.
